# The role of ambulatory blood pressure monitoring in enhancing medication adherence among patients with newly diagnosed hypertension: an analysis of the National Health Insurance cohort database

**DOI:** 10.1186/s40885-024-00264-x

**Published:** 2024-03-01

**Authors:** Hack-Lyoung Kim, So-Jeong Park, Yoon-Jong Bae, Sang Hyum Ihm, Jinho Shin, Kwang-Il Kim

**Affiliations:** 1 Division of Cardiology, Department of Internal Medicine, Boramae Medical Center, Seoul National University College of Medicine, Seoul, Republic of Korea; 2 Department of Data Science, Hanmi Pharm. Co., Ltd., Seoul, Republic of Korea; 3 Division of Cardiology, Department of Internal Medicine, Bucheon St. Mary’s Hospital & Catholic Research Institute for Intractable Cardiovascular Disease, College of Medicine, The Catholic University of Korea, Seoul, Republic of Korea; 4 Division of Cardiology, Department of Internal Medicine, Hanyang University College of Medicine, Seoul, Republic of Korea; 5 Department of Internal Medicine, Seoul National University Bundang Hospital, Seoul National University College of Medicine, Seongnam, Republic of Korea

**Keywords:** Adherence, Antihypertensive drugs, Ambulatory blood pressure monitoring, Hypertension

## Abstract

**Background:**

Improving adherence to antihypertensive medication (AHM) is a key challenge in hypertension management. This study aimed to assess the impact of ambulatory blood pressure monitoring (ABPM) on AHM adherence.

**Methods:**

We utilized the Korean National Health Insurance Service database. Among patients newly diagnosed with hypertension who started AHM between July 2010 and December 2013, we compared clinical characteristics and adherence between 28,116 patients who underwent ABPM prior to starting AHM and 118,594 patients who did not undergo ABPM. Good adherence was defined as a proportion of days covered (PDC) of 0.8 or higher.

**Results:**

The total study population was 146,710, with a mean age of 50.5 ± 6.4 years; 44.3% were female. Co-morbidities were noted in 4.2%. About a third of patients (33.1%) showed good adherence. The ABPM group had a notably higher PDC (total PDC: 0.64 ± 0.35 *vs*. 0.45 ± 0.39; *P* < 0.001), irrespective of the number of medications, dosing frequency, or prescription duration. After adjusting for significant clinical variables, ABPM was still closely linked with good adherence (odds ratio, 2.35; 95% confidence interval, 2.28–2.41; *P* < 0.001).

**Conclusions:**

In newly diagnosed hypertension, undergoing ABPM prior to AHM prescription appears to enhance adherence to AHM. The exact mechanisms driving this association warrant further exploration.

**Graphical Abstract:**

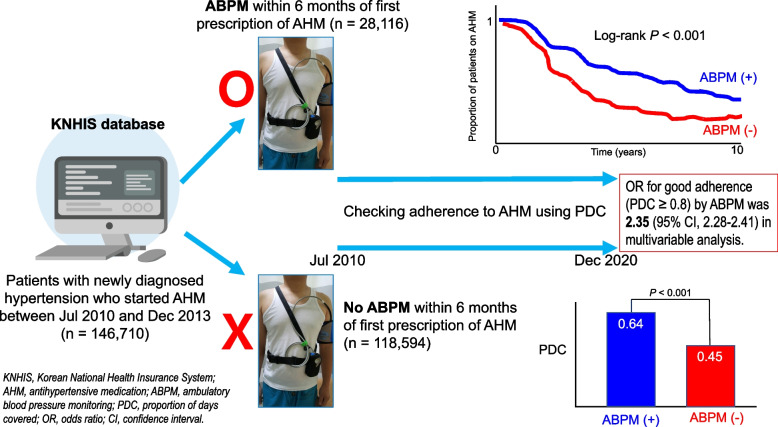

## Introduction

Hypertension is a leading global health challenge due to its high prevalence and strong association with cardiovascular complications such as heart attacks, strokes, kidney diseases, and heart failure [1]. Thus, controlling hypertension is crucial for an individual’s overall health and longevity [2, 3]. Adherence to prescribed antihypertensive medication (AHM) plays a pivotal role in this endeavor. By taking AHM as directed, individuals can achieve and maintain optimal blood pressure (BP) levels, significantly reducing the risks associated with uncontrolled hypertension [4–7]. Moreover, medication adherence helps healthcare providers accurately assess treatment effectiveness, allowing them to make necessary adjustments in a timely manner [8]. Non-adherence, on the other hand, can lead to uncontrolled hypertension, creating a false perception of treatment failure and prompting unnecessary changes in medication [9]. Moreover, non-adherence is associated with poor clinical outcomes [10, 11]. It’s also noteworthy that medication adherence helps in reducing healthcare costs, particularly those associated with hospitalization and emergency care for hypertensive crises and related complications [12, 13]. Thus, medication adherence is pivotal to successful hypertension management and improving overall patient outcomes [4].

Ambulatory blood pressure monitoring (ABPM) is essential for diagnosis and monitoring in hypertension [14–16]. Unlike traditional single-time measurements, ABPM records BP at regular intervals over 24 h, capturing day-to-night fluctuations. This comprehensive profiling helps identify conditions like white-coat hypertension and masked hypertension [14]. It also provides a more accurate assessment of cardiovascular risk [17, 18], given its ability to measure nocturnal BP [19]. Recently, the importance of ABPM has been increasingly emphasized in the hypertension management [20–22].

Several strategies have been employed to enhance medication adherence in hypertensive patients [8], yet it remains notably low [23]. While numerous reports suggest that home BP measurements can increase patients’ medication adherence [24, 25], the influence of ABPM on medication adherence remains unreported. In this study, we investigated whether conducting ABPM before prescribing AHM could enhance patients’ adherence to their AHM.

## Methods

### Study patients and protocol

**Fig. 1:**
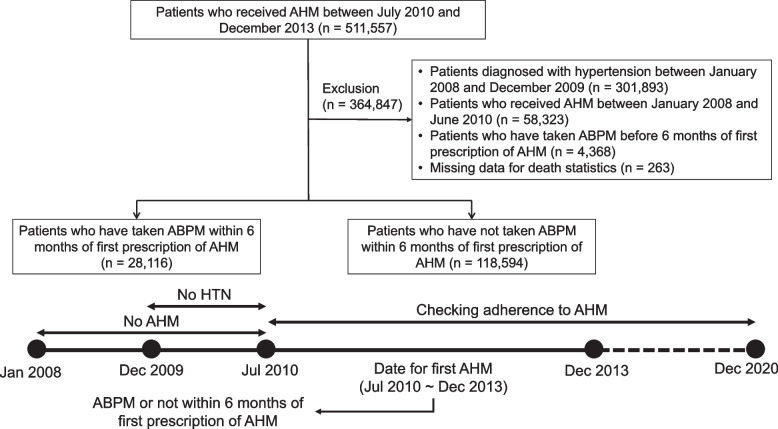
Study flow chart for patient enrollment. AMH, antihypertensive medication; ABPM, ambulatory blood pressure monitoring; HTN, hypertension

We utilized the Korean National Health Insurance Service database. Among 511,557 patients diagnosed with hypertension (International Classification of Diseases [ICD] codes I10-I15) and who started AHM between July 2010 and December 2013, patient in whom ABPM (prescription code: E6547) performed 6 months or more before the first AHM prescription (*n* = 4,368), and patients with missing death statistics data (*n* = 263) were excluded. To focus on newly prescribed AHM hypertension patients, we excluded those diagnosed with hypertension between January 2008 and December 2009 (*n* = 30,1893) and any patients prescribed with AHM between January 2009 and June 2010 (*n* = 58,323), as depicted in Fig. 1. The ABPM group comprised 28,116 patients who had ABPM conducted within 6 months before the date when AHM was first prescribed. The non-ABPM group consisted of 118,594 patients who had not taken ABPM. Study protocol was reviewed by Institutional Review Board (IRB) in Hanyang University Seoul Hospital (HYUH 2020–02-009) and approved. Informed consent was waived by the IRB.

### Clinical data

Information on age, sex, diagnoses of diabetes mellitus (E10-E14), dyslipidemia (E78), heart failure (I50), ischemic heart disease (I20, I25), chronic kidney disease (N18, I13.0, I13.2), atrial fibrillation (I48), ischemic stroke (I63, I64), intracranial hemorrhage (I60, I61, I62), and myocardial infarction (I21, I22), along with prescriptions for anti-platelet drugs, anti-diabetic drugs and statins were available. Co-morbidities were identified solely by ICD codes. Ischemic heart disease includes angina pectoris (I20), atherosclerotic heart disease (I25.1), old myocardial infarction (I25.2), ischemic cardiomyopathy (I25.5), and other forms of chronic ischemic heart disease (I25.8, I25.9). Myocardial infarction encompasses acute myocardial infarction (I21), which refers to heart attacks that have occurred recently, typically within the past 28 days, and subsequent myocardial infarction (I22), denoting the occurrence of a new heart attack shortly after an initial event.

### Medication adherence

Good adherence was defined by a proportion of days covered (PDC) of 0.8 or higher. The PDC was calculated as follows: (the number of days covered by the prescription drugs) ÷ (the number of days in period) [26, 27]. Measurements of PDC were taken from the start of AHM until December 2020 (Fig. 1).

### Statistical analysis

Continuous variables are presented as mean ± standard deviation, while categorical variables are denoted as n (%). For comparisons between groups, continuous variables were assessed using Student’s *t*-test, and categorical variables were analyzed using the chi-square test with Yates continuity correction. Stepwise logistic regression evaluated the association between good adherence and various covariates. The PDC comparisons between the two groups were conducted using two-way analysis of variance, Student’s t-test. Drug adherence estimation employed the Kaplan–Meier method, with intergroup comparisons assessed via the log-rank test. The associations between ABPM and PDC were examined using a logistic regression with stepwise selection, adjusted for variables that showed a significant difference between the study groups (*P* value < 0.1). Results are presented as odds ratio (OR) with a 95% confidence interval (CI). All statistical analyses were performed using SAS Enterprise Guide version 9.4 (SAS Institute Inc., Cary, NC, USA).

## Results

The characteristics of study patients, differentiated by the presence or absence of ABPM, are detailed in Table 1. Patients who underwent ABPM were younger (49.32 ± 12.52 *vs*. 56.52 ± 13.72 years; *P* < 0.001) and had a slightly higher proportion of women (46.63% *vs*. 46.43%; *P* < 0.001) compared to those without ABPM. Additionally, patients with ABPM had a higher prevalence of risk factors like dyslipidemia, atrial fibrillation, and ischemic heart disease. In contrast, occurrences of ischemic stroke, intracranial hemorrhage, and transient ischemic attack were more common in patients not subjected to ABPM. Medications such as anti-platelets, anti-diabetics, and statins were prescribed less frequently to those with ABPM than to those without.

**Table 1:** Characteristics of study in patients with and without ABPM

Characteristic	Without ABPM (*n* = 118,594)	With ABPM (*n* = 28,116)	*P* value
*Age, years*	56.52 ± 13.72	49.32 ± 12.52	< 0.001
~ 29	3,317 (2.80)	1,860 (6.62)	< 0.001
30 ~ 39	8,609 (7.26)	3,767 (13.40)	
40 ~ 49	22,315 (18.82)	7,882 (28.03)	
50 ~ 59	37,249 (31.41)	9,080 (32.29)	
60 ~ 69	25,993 (21.92)	4,088 (14.54)	
70 ~ 79	15,308 (12.91)	1,292 (4.60)	
80 ~	5,803 (4.89)	147 (0.52)	
Female sex	54,709 (46.13)	12,266 (46.63)	< 0.001
*Comorbidities*			
Diabetes mellitus	14,550 (12.27)	3,530 (12.56)	0.192
Dyslipidemia	22,491 (18.96)	8,650 (30.77)	< 0.001
Chronic kidney disease	269 (0.23)	62 (0.22)	0.896
Atrial fibrillation	555 (0.47)	191 (0.68)	< 0.001
Ischemic heart disease	3,811 (3.21)	1,585 (5.64)	< 0.001
Myocardial infarction	251 (0.21)	56 (0.21)	1.000
Heart failure	556 (0.47)	130 (0.46)	0.925
Ischemic stroke	2,683 (2.26)	427 (1.52)	< 0.001
Intracranial hemorrhage	315 (0.27)	51 (0.18)	0.013
Transient ischemic attack	1,238 (1.04)	253 (0.90)	0.033
*Concomitant medications*			
Anti-platelets	6,842 (5.77)	1,249 (4.44)	< 0.001
Anti-diabetics	8,078 (6.81)	841 (2.99)	< 0.001
Statins	9,746 (8.22)	2,164 (7.70)	0.004

Numbers are expressed as mean ± standard deviation or n (%)

*ABPM* ambulatory blood pressure monitoring

Approximately one-third of patients (48,673/146,980 = 33.11%) demonstrated good adherence. The characteristics distinguishing patients with good and poor adherence are outlined in Table 2. Patients with good adherence were more frequently subjected to ABPM compared to those with poor adherence (27.50% *vs*. 15.02%; *P* < 0.001). Those with good adherence tended to be older (56.24 ± 12.11 *vs*. 54.59 ± 14.53 years; *P* < 0.001) and had a slightly lower female representation (44.86% *vs*. 46.04%; *P* < 0.001). Good adherence was associated with a higher prevalence of comorbidities, including diabetes mellitus, dyslipidemia, atrial fibrillation, ischemic heart disease, myocardial infarction, and ischemic stroke. Medications like anti-platelets, anti-diabetics, and statins were more commonly prescribed to patients with good adherence. There was no significant variance in the number of AHM prescriptions between the two groups (1.83 ± 2.25 *vs*. 1.82 ± 2.82; *P* = 0.539). However, the daily dosing frequency of AHM was slightly lower for those with good adherence (1.28 ± 0.63 *vs*. 1.39 ± 0.80; *P* < 0.001).

**Table 2:** Characteristics of study patients according to drug adherence

Characteristic	Poor adherence (*n* = 98,307)	Good adherence (*n* = 48,673)	*P* value
With ABPM	14,730 (15.02)	13,386 (27.50)	< 0.001
*Age, years*	54.59 ± 14.53	56.24 ± 12.11	< 0.001
~ 29	4,576 (4.67)	601 (1.23)	< 0.001
30 ~ 39	9,288 (9.47)	3,088 (6.34)	
40 ~ 49	20,067 (20.47)	10,130 (20.81)	
50 ~ 59	29,893 (30.49)	16,436 (33.77)	
60 ~ 69	18,666 (19.04)	11,415 (23.45)	
70 ~ 79	10,999 (11.22)	5,601 (11.51)	
80 ~	4,548 (4.64)	1,402 (2.88)	
Female sex	45,140 (46.04)	21,835 (44.86)	< 0.001
*Comorbidities*			
Diabetes mellitus	11,583 (11.81)	6,497 (13.35)	< 0.001
Dyslipidemia	20,233 (20.43)	11,108 (22.82)	< 0.001
Chronic kidney disease	226 (0.23)	105 (0.22)	0.614
Atrial fibrillation	455 (0.46)	291 (0.60)	< 0.001
Ischemic heart disease	3,811 (3.21)	1,585 (5.64)	0.001
Myocardial infarction	183 (0.19)	127 (0.26)	0.004
Heart failure	447 (0.46)	239 (0.49)	0.375
Ischemic stroke	2,005 (2.05)	1,105 (2.27)	0.005
Intracranial hemorrhage	242 (0.25)	124 (0.25)	0.817
Transient ischemic attack	1,009 (1.03)	482 (0.99)	0.501
*Concomitant medications*			
Anti-platelets	5,100 (5.20)	2,991 (6.15)	< 0.001
Anti-diabetics	5,617 (5.73)	3,302 (6.78)	< 0.001
Statins	7,539 (7.69)	4,371 (8.98)	< 0.001
*AHM number*	1.82 ± 2.82	1.83 ± 2.25	0.539
0 ~ < 1 pill	9,430 (9.62)	3,804 (7.82)	< 0.001
1 pill	35,277 (35.98)	15,197 (31.22)	
1 ~ < 2 pills	29,120 (29.70)	17,570 (36.10)	
2 ~ < 3 pills	14,405 (14.69)	7,341 (15.08)	
3 ~ < 4 pills	4,198 (4.28)	1,946 (4.00)	
≥ 4 pills	5,607 (5.72)	2,815 (5.78)	
*Daily dosing frequency of AHM*	1.39 ± 0.80	1.28 ± 0.63	< 0.001
≤ 1 pill	52,889 (53.95)	29,692 (61.00)	< 0.001
1 < ~ < 2 pills	25,129 (25.63)	13,335 (27.40)	
≥ 2 pills	20,019 (20.42)	5,646 (11.60)	

Numbers are expressed as mean ± standard deviation or n (%). ABPM, ambulatory blood pressure monitoring

*AHM* anti-hypertensive medication

PDC of patients with and without ABPM are shown in Table 3. PDC was significantly higher in the ABPM group (total PDC, 0.64 ± 0.35 *vs*. 0.45 ± 0.39; *P* < 0.001), regardless of the number of medications, dosing frequency, and prescription duration (*P* < 0.05 for each). The proportion of the patients with PDC ≥ 0.8 was significantly higher in patients with ABPM than those without ABPM (47.60% vs 27.47%; *P* < 0.001).

**Table 3:** PDC according to medications

Characteristic	Without ABPM (*n* = 118,594)	With ABPM (*n* = 28,116)	*P* value
*PDC, total*	0.45 ± 0.39	0.64 ± 0.35	< 0.001
*PDC* ≥ *0.8*	35,287 (27.47)	13,386 (47.60)	< 0.001
*PDC according to AHM number*			
0 ~ < 1 pill	0.41 ± 0.37	0.61 ± 0.34	< 0.001
1 ≤ ~ < 2 pills	0.45 ± 0.39	0.64 ± 0.36	
2 ~ < 3 pills	0.48 ± 0.39	0.65 ± 0.35	
3 ~ < 4 pills	0.47 ± 0.39	0.59 ± 0.38	
≥ 4 pills	0.51 ± 0.37	0.58 ± 0.35	
*PDC according to AHM number and duration*^a^			
0 ~ < 1 pill	0.36 ± 0.35	0.55 ± 0.35	< 0.001
1 ~ < 2 pills	0.93 ± 0.12	0.97 ± 0.07	
2 ~ < 3 pills	0.92 ± 0.14	0.97 ± 0.06	
3 ~ < 4 pills	0.91 ± 0.18	0.91 ± 0.18	
≥ 4 pills	0.83 ± 0.24	0.95 ± 0.09	
*PDC according to the number of all drugs*			
1 ~ < 5 pills	0.47 ± 0.39	0.64 ± 0.36	< 0.001
5 ~ 10 pills	0.51 ± 0.37	0.66 ± 0.33	
≥ 10 pills	0.34 ± 0.38	0.55 ± 0.38	
*PDC according to the daily dosing frequency of AHM*			
≤ 1 pill	0.37 ± 0.38	0.61 ± 0.36	< 0.001
1 < ~ < 2 pills	0.59 ± 0.36	0.75 ± 0.29	
≥ 2 pills	0.62 ± 0.36	0.71 ± 0.31	

Numbers are expressed as mean ± standard deviation or n (%)

^a^[(tablet number × sum of days supplied)/number of days]

*PDC* proportion of days covered, *ABPM* ambulatory blood pressure monitoring, *AHM* anti-hypertensive medication

**Fig. 2:**
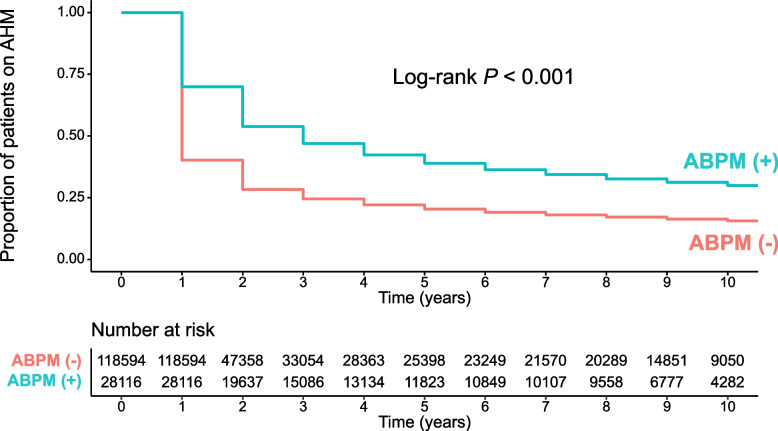
Drug adherence in patients with and without ABPM. AMH, antihypertensive medication; ABPM, ambulatory blood pressure monitoring

Table 4 demonstrates the factors associated with good adherence as revealed by multivariable binary logistic regression analysis. Conducting ABPM was significantly associated with good adherence even after accounting for important clinical covariates (OR, 2.35; 95% CI, 2.28–2.41; *P* < 0.001). Compared to individuals in their 60 s, those in the age groups both younger and older than 60 demonstrated poorer adherence. Factors associated with poor adherence also included dyslipidemia, taking fewer than one medication (in comparison to one medication), and a higher daily dosing frequency of more than once a day. Conversely, a history of ischemic heart disease and myocardial infarction, the use of anti-platelets, anti-diabetics, and statins, as well as an AHM count greater than one, were independent predictors of good adherence. Figure 2 displays a significant time-specific difference in drug adherence based on the implementation of ABPM, as evidenced by the Kaplan–Meier curve (log-rank *P* < 0.001).

**Table 4:** Multivariable binary logistic regression analysis showing factors associated with good adherence

Variable	OR (95% CI)	*P* value
With ABPM	2.35 (2.28–2.41)	< 0.001
*Age, years*		
~ 29	0.18 (0.16–0.19)	< 0.001
30 ~ 39	0.48 (0.45–0.50)	< 0.001
40 ~ 49	0.76 (0.73–0.79)	< 0.001
50 ~ 59	0.88 (0.85–0.90)	< 0.001
60 ~ 69	1 (reference)	-
70 ~ 79	0.85 (0.82–0.89)	< 0.001
80 ~	0.52 (0.49–0.56)	< 0.001
Dyslipidemia	0.96 (0.93–0.99)	0.015
Ischemic heart disease	1.07 (1.01–1.13)	0.035
Myocardial infarction	1.30 (1.03–1.65)	0.028
Anti-platelets	1.07 (1.02–1.13)	0.009
Anti-diabetics	1.15 (1.10–1.21)	< 0.001
Statins	1.10 (1.05–1.16)	0.001
*AHM number*		
0 ~ < 1	0.89 (0.85–0.93)	< 0.001
1	1 (reference)	-
1 ~ < 2	1.82 (1.77–1.88)	< 0.001
2 ~ < 3	2.09 (2.01–2.18)	< 0.001
3 ~ < 4	2.29 (2.15–2.44)	< 0.001
≥ 4	2.67 (2.15–2.44)	< 0.001
*Daily dosing frequency of AHM*		
≤ 1	1 (reference)	
1 < ~ < 2	0.67 (0.65–0.69)	< 0.001
≥ 2	0.35 (0.34–0.37)	< 0.001

*ABPM* ambulatory blood pressure, *AHM* anti-hypertensive medication, *OR* odds ratio, *CI* confidence interval

## Discussion

Using a substantial cohort of newly diagnosed hypertensive patients from the national claims database, we aimed to determine whether undergoing ABPM enhances AHM adherence. The primary findings of our study are: 1) only 33.11% of patients exhibited good adherence, 2) PDC values were significantly higher in the ABPM group, regardless of the number of medications or dosing frequency, and 3) the act of undergoing ABPM emerged as a significant predictor of good adherence, even after accounting for essential clinical variables. To the best of our knowledge, this is the first study specifically examining the relationship between ABPM and adherence to AHM.

Medication adherence to AHM directly influences a patient’s prognosis [5, 10–12], making it crucial for hypertension treatment. While numerous methods have been proposed through various studies to enhance medication adherence to AHM, their effects have been limited [8]. Our research indicates that administering ABPM before prescribing AHM enhances patients’ adherence to AHM. Many studies suggest that home BP measurements can improve patients’ adherence to AHM [24, 25]. However, even though ABPM is pivotal for diagnosing and monitoring hypertension [14], it remains uncertain if ABPM can improve patients’ adherence to AHM. Given these insights, our study presents a novel perspective.

The mechanism by which ABPM promotes adherence to AMH remains unclear, but several hypotheses have been proposed. Primarily, ABPM gives a more comprehensive view of a patient’s BP status [14–16]. When patients view their ABPM results, getting more understanding the fluctuating nature of the blood pressure and the representativeness of average BP, they may be more determined to follow medication regimens and make necessary lifestyle changes, given the real-time data and trends highlighting the effects of their actions and medications. Furthermore, ABPM is a valuable tool for dose adjustments even though repeated measurement is quite limited by discomfort during ABPM [16]. By measuring BP throughout the day, clinicians can gauge the efficacy of medications and tweak doses to optimize BP management. ABPM also serves as an educational platform for healthcare providers to inform patients about daily BP fluctuations, the significance of medication consistency, and how lifestyle decisions influence BP. Identifying phenomena like white-coat hypertension with ABPM ensures patients are not overmedicated, enhancing adherence by preventing unwarranted drug side effects and costs [28]. On the other hand, recognizing masked hypertension can emphasize the need for consistent medication adherence and ensure that the most appropriate treatment is prescribed [29]. Additionally, wearing a monitoring device might strengthen patients’ awareness of their health metrics, fostering a more profound commitment to their medication regimen. This blend of accurate diagnosis, patient involvement, and immediate feedback underscores the critical role ABPM assumes in improving medication adherence in hypertensive patients. Approaches to improve feasibility for repeated ABPM such as wearable device might take advantage of the cognitive and behavioral aspects by ABPM much more easily. But, first of all, the validation issues needs to be solved [30].

In our study, apart from considering whether ABPM was performed, we observed heightened medication adherence among participants in their 60 s, especially those with multiple comorbidities such as myocardial infarction and ischemic heart disease and those on numerous concurrent medications. This trend might be more reflective of Korean cultural characteristics than any unique pathophysiological aspects. Among younger individuals, there is a diminished awareness of hypertension [11], likely owing to the demands of their professional lives. After retirement, individuals in their 60 s tend to place more emphasis on health, adopt healthier lifestyles, and adhere to medications. As age advances, the prevalence of diseases rises, leading to an increase in the number of medications prescribed. It is understood that individuals who have a disease and are on medication for it tend to be more attentive to their health and exhibit better medication adherence compared to those without such conditions [31]. Beyond the age of 70, medication adherence appears to decline, potentially due to cognitive impairments or challenges with mobility [32]. While it’s generally accepted that there is an inverse relationship between the number of prescribed medications and patient adherence [33], our study presented contrary findings. This deviation could be attributed to a significant segment of the older population being health-conscious with more medications, resulting in better adherence to medication regimens. Our study also found that the number of times a drug was taken daily was inversely related to the patient’s adherence to the medication regimen, consistent with prior reports [34]. It reiterates that reducing the frequency of AHM intake can effectively enhance medication adherence.

ABPM is recommended as the gold standard for diagnosing and monitoring hypertension [20–22]. Based on our findings, subsequent research should investigate how much ABPM enhances patient adherence. Should further evidence from these investigations bolster our findings, the role of ABPM as an essential tool to boost adherence will be solidified. However, despite its evident advantages, there are obstacles to the universal adoption of ABPM. Factors such as the device’s comfort and usability, cost, and overall accessibility can hinder its widespread use [16]. It is imperative that future research address these challenges and devise strategies to make ABPM more available and appealing to users. With the broader implementation of ABPM, these inherent limitations will likely pose less of an obstacle to its adoption. While our study offers promising results on the benefits of ABPM in promoting medication adherence, it would be essential to assess the effects on cardiovascular outcome. Future research could explore whether these positive adherence trends influence the overall prognosis of hypertensive patients.

Our studies present somewhat conflicting and mixed results, particularly regarding age and risk factors. Notably, patients who underwent ABPM were younger but exhibited a higher number of cardiovascular risk factors compared to those who did not undergo ABPM. Conversely, patients with good medication adherence were older and also had more risk factors than those with poor adherence. This suggests that while high-risk younger patients were more proactive in undergoing ABPM testing, their adherence to medication was lower. In contrast, older patients, particularly those in their 60 s, had more risk factors but demonstrated better medication adherence.

### Study limitations

We acknowledge several limitations in our study. Firstly, the retrospective nature of our analysis may introduce selection bias. Table 1 shows notable clinical characteristic differences exist between patients who underwent ABPM and those who did not. Propensity score matching analysis could have yielded more reliable and valid results, but it was not feasible in our study. Nevertheless, we have endeavored to adjust for these disparities as comprehensively as possible via multivariable logistic regression analysis. Secondly, our study did not account for other potential determinants of medication adherence, such as socioeconomic status (e.g., education or income levels) or the use of fixed-dose combination drugs. Thirdly, our reliance on national database data might introduce biases from overlooked records, misclassifications, or coding inaccuracies. Fourthly, our adherence assessment hinges on the PDC threshold of 0.8 or higher, which may not accurately reflect the nuances of medication adherence: simply possessing medication does not guarantee its appropriate use. Fifthly, our data spans from 2010–2013, which might not encapsulate contemporary medical practices or evolving patient behaviors. Lastly, our research exclusively centers on whether ABPM was conducted within six months preceding the prescription of AHM for newly diagnosed hypertension patients, limiting the generalizability of our findings to other demographics.

## Conclusions

We showed that undertaking ABPM before initiating AHM in newly diagnosed hypertensive patients notably enhanced adherence to the medication regimen. Our findings underscore the potential utility of ABPM in bolstering adherence to AHM in hypertensive patients. Subsequent studies are essential to validate our observations and to elucidate the clinical outcomes influenced by ABPM.

## Data Availability

The data supporting the findings of this study are available from the Korean National Health Insurance Service (KNHIS). However, restrictions apply to the accessibility of these data. Nonetheless, the data can be made available by the authors upon reasonable request, subject to permission from the KNHIS.

## Abbreviations

ABPM: Ambulatory blood pressure monitoring
AHM: Antihypertensive medication
BP: Blood pressure
CI: Confidence interval
ICD: International Classification of Diseases
IRB: Institutional review board
OR: Odds ratio
PDC: Proportion of days covered
